# Identification of *Helianthus annuus* allergens in subjects with allergy to sunflower

**DOI:** 10.1186/2045-7022-4-S2-P14

**Published:** 2014-03-17

**Authors:** Mª Luisa Macias, Francisca Gomez, Ana Aranda, Natalia Blanca-Lopez, Cristobalina Mayorga, Mª Jose Torres, Gabriela Canto, Araceli Diaz-Perales, Miguel Blanca

**Affiliations:** 1 Carlos Haya Hospital-IBIMA, Research Laboratory, Malaga, Spain; 2 Carlos Haya Hospital, Allergy Service, Malaga, Spain; 3 Infanta Leonor Hospital, Allergy Service, Madrid, Spain; 4 Plant Biotechnology Institute, UPM-INIA, Madrid, Spain

## Background

Sunflower seeds (Helianthus annuus) can trigger anaphylactic reactions, generalized urticaria, angioedema, oral allergy syndrome and other symptoms after ingestion. These reactions have been attributed to 2S albumins (SFA-8) and LTP (Hel a 3). We aimed to characterize the basophil response to storage proteins and oleosins from sunflower seed in patients allergic to sunflower.

## Methods

The proteins 2S, 11S and oleosins were purified from a raw sunflower seed extract by FPLC/HPLC and identified by specific antibodies and peptide mass fingerprinting. We tested the immunological recognition of these proteins by basophil activation test (BAT). Four concentrations (1, 0.2, 0.1, 0.02 **ì**g/ml) of each protein and the sunflower roasted extract were used. Ten patients were selected by clinical history and skin prick test positive to commercial extract. Twelve subjects with skin prick test negative to commercial extract and not food allergy were included as controls.

## Results

All patients showed a positive basophil response to roasted extract. BAT was positive in 87.5% of cases for 2S albumin, 60% for oleosins, and 57.14% for 11S albumin. 50% of patients were positive to the 3 proteins, 37.5% only for 2S albumin and 12.5% for storage proteins (both 2S and 11S albumin). In 40% of controls the concentration 1 **ì**g/ml of 2S albumin induced low basophil activation.

## Conclusions

All the sunflower allergens tested in our group of patients were able to induce basophil activation in a high percentage of cases. Storage proteins and oleosins are responsible for sunflower allergy in 50% of cases.

